# Local environment‐driven adaptive evolution in a marine invasive ascidian (*Molgula manhattensis*)

**DOI:** 10.1002/ece3.7322

**Published:** 2021-03-06

**Authors:** Yiyong Chen, Yangchun Gao, Xuena Huang, Shiguo Li, Aibin Zhan

**Affiliations:** ^1^ Research Center for Eco‐Environmental Sciences Chinese Academy of Sciences Haidian District Beijing China; ^2^ University of Chinese Academy of Sciences Chinese Academy of Sciences Shijingshan District Beijing China; ^3^ Guangdong Key Laboratory of Animal Conservation and Resource Utilization Institute of Zoology Guangdong Academy of Sciences Haizhu District Guangzhou China

**Keywords:** adaptive evolution, biological invasion, population genomics, RADseq, salinity adaptation

## Abstract

Elucidating molecular mechanisms of environment‐driven adaptive evolution in marine invaders is crucial for understanding invasion success and further predicting their future invasions. Although increasing evidence suggests that adaptive evolution could contribute to organisms’ adaptation to varied environments, there remain knowledge gaps regarding how environments influence genomic variation in invaded habitats and genetic bases underlying local adaptation for most marine invaders. Here, we performed restriction‐site‐associated DNA sequencing (RADseq) to assess population genetic diversity and further investigate genomic signatures of local adaptation in the marine invasive ascidian, *Molgula manhattensis*. We revealed that most invasive populations exhibited significant genetic differentiation, low recent gene flow, and no signal of significant population bottleneck. Based on three genome scan approaches, we identified 109 candidate loci potentially under environmental selection. Redundancy analysis and variance partitioning analysis suggest that local environmental factors, particularly the salinity‐related variables, represent crucial evolutionary forces in driving adaptive divergence. Using the newly developed transcriptome as a reference, 14 functional genes were finally obtained with potential roles in salinity adaptation, including SLC5A1 and SLC9C1 genes from the solute carrier gene (SLC) superfamily. Our findings confirm that differed local environments could rapidly drive adaptive divergence among invasive populations and leave detectable genomic signatures in marine invaders.

## Graphical abstract

We investigated genomic signatures of rapid local adaptation in the highly invasive ascidian, Molgula manhattensis. We found that local environmental factors, such as salinity‐related variables, represented crucial evolutionary forces in driving adaptive divergence during biological invasions. We also identified functional genes responsible for salinity adaptation, such as SLC5A1 and SLC9C1 genes from the solute carrier gene (SLC) superfamily.

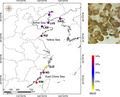

## 1 INTRODUCTION

With rapid global climate change and frequent anthropogenic activities, biological invasion has become a worldwide threat to marine and coastal ecosystems (N’Guyen et al., 2016; Papacostas et al., 2017). Marine invasive species, which are commonly introduced by multiple vectors such as shipping and aquaculture, have rapidly colonized and spread into a wide range of coastal regions and caused severe ecological and economic losses (Chen et al., 2017; Lins et al., 2018). When expanding into novel environments, introduced populations inevitably encounter a variety of novel abiotic and biotic constraints, and such multiple stressors have been proposed to potentially drive genetic divergence among populations (Moran & Alexander, 2014; White et al., 2013). Additionally, both the rate and range of environmental changes during biological invasions are often orders of magnitude faster and greater than what species experience in natural processes (e.g., seasonal fluctuations) in their native habitats (Lin et al., 2017). In order to cope with various environmental challenges, some invasive species have considerable potentials for rapid adaptation (Batz et al., 2020; Lee, 2002). Such a common feature indicates that invasive species can provide promising materials to investigate how organisms adapt to changing environments (Bock et al., 2015; Zhan et al., 2015). Indeed, empirical studies have recently evidenced that environment‐driven microevolution, such as local adaptation, could play an important role in organisms’ adaptation to different environments (Bock et al., 2015; Ni et al., 2018). However, there remain knowledge gaps regarding local adaptation in marine invaders in their invasive range and genetic/genomic bases underlying local adaptation have not well been elucidated (Tepolt, 2015). In particular, it remains largely unexplored how local environmental conditions influence population genetic variation in a set of invasive populations. A better understanding of genetic/genomic bases of environmental adaptation will provide useful insights into evolutionary dynamics of invasive species in response to environmental changes, as well as predict their future invasion dynamics into new habitats (Jeffery et al., 2018; Prentis et al., 2008).

Marine organisms, especially sessile tunicates, are more vulnerable to be influenced by local environmental challenges, mainly owing to their sessile lifestyle (see the review by Zhan et al., 2015). Different local environmental factors, such as water salinity, temperature, and dissolved oxygen stressors, can greatly affect their growth, development, reproduction, and geographical distributions. Environmental heterogeneity could potentially lead to genetic divergence among populations, and thus investigating genomic signatures imposed by local environments would provide insights into mechanisms and dynamics of environmental adaptation (Attard et al., 2018; Hecht et al., 2015). However, it remains challenging to disentangle the effects of environmental selection from population demographic history in wild populations (Ferchaud & Hansen, 2016), mainly because population demography‐associated processes, such as genetic drift and gene flow, are often accompanied by natural selection and further may obscure, or even eliminate, signals of natural selection in introduced populations (Li et al., 2012; Lotterhos & Whitlock, 2015). Genetic drift may contribute to random changes of allele frequency, further complicating the identification of loci under selection (Lotterhos & Whitlock, 2014). For many sessile tunicates, although they have limited natural dispersal ability owing to short pelagic larval durations and sessile adults, human‐mediated gene flow is likely to drive genetic homogeneity (Chen et al., 2017; Zhan et al., 2010). Thus, it poses challenges for the accurate estimation of population genetic divergence when merely using "low‐resolution" genetic markers (e.g., a limited number of microsatellites). Additionally, spatial distributions may confound the environmental effect in the analysis of local adaptation if environmental factors are spatially autocorrelated (Benestan et al., 2016; Rellstab et al., 2015). It is therefore important to choose a promising model system and state‐of‐the‐art techniques to investigate the mechanisms of local adaptation in marine invasive species.

The notoriously invasive ascidian, *Molgula manhattensis*, which is native to the east coast of North America, has invaded large geographical scales globally and caused significantly negative ecological, environmental, and economic impacts in invaded marine and coastal ecosystems (Chen, Li, et al., 2017; Haydar et al., 2011). In China, *M. manhattensis* was firstly recorded at Tanggu Harbor in Tianjin in 1976 (Zheng, 1995). Since then, it has widely spread along the Chinese coast, especially in Bohai and Yellow seas, where this species has been reported as the primary fouling organism and caused huge damages (Chen, Li, et al., 2017). Owing to its biological features including a short pelagic larval phrase and sessile adults as well as a high self‐recruitment rate, this species is characterized by a low natural dispersal ability, and their range expansion in China is likely to be transported with aquaculture transfers and/or *via* shipping as fouling organisms. Across its wide distribution range along the Chinese coast (spanning > 15 degrees of latitude), this species has encountered heterogeneous environmental conditions, especially water temperature and salinity, which may impose strong selective pressures during range expansions. In addition, some biological characteristics, such as high fecundity, rapid growth rate, and short life cycle, can facilitate this ascidian to rapidly colonize in novel environments (Chen, Li, et al., 2017). Altogether, the rapid and widespread colonization by *M. manhattensis* along the Chinese coast provides us a promising model system to study mechanisms of local adaptation driven by divergent local environments, particularly by water temperature and salinity.

As shown in one previous study with one mitochondrial COI marker and 12 nuclear microsatellites, they revealed weak genetic structure at large geographical scales but significant genetic differentiation among some neighboring *M. manhattensis* populations along the Chinese coast (Chen, Li, et al., 2017). We propose the effect of local environmental selection on significant population genetic differentiation, but direct evidence remained unavailable, owing to the limited number of genetic markers used. In addition, the observed inconsistent results of population genetic diversity by mtDNA and nuclear markers were likely derived from their different inheritance natures responding to natural selection (Chen, Li, et al., 2017). As suggested by a growing number of related studies, the genome‐wide survey would provide more power in uncovering subtle population genetic structure (Benestan et al., 2015; Blanco‐Bercial & Bucklin, 2016) and more importantly detecting signatures of environment‐driven local adaptation (Sandoval‐Castillo et al., 2018; Waterhouse et al., 2018). With technical advances in next‐generation sequencing technologies, restriction‐site‐associated DNA sequencing (RADseq) provides a relatively economical and efficient approach for genome‐wide genotyping of DNA polymorphisms (Andrews et al., 2016). Population genomics offer an opportunity to investigate genetic structure and gene flow more accurately and comprehensively, further contributing to the differentiation of locus‐specific effects (e.g., selection, mutation) from genome‐wide effects (e.g., gene flow, genetic drift, bottleneck) (Luikart et al., 2003). In addition, the utilization of this robust genotyping method, as well as efficient theoretical and analytical approaches such as appropriate genome scan methods, can contribute to the investigation of genomic basis of local adaptation, particularly the relative contribution of environmental factors to population genetic divergence (de Villemereuil et al., 2014; François et al., 2016).

Using the RADseq on *M. manhattensis* populations collected from a wide geographical range along the Chinese coast, we aim to test the hypothesis that differed local environmental factors could drive adaptive genetic divergence among populations and leave detectable signatures in the genome of marine invaders. Based on a neutral single nucleotide polymorphisms (SNPs) dataset, we firstly assessed population genetic variation and gene flow patterns. Subsequently, we combined population differentiation (PD) and environmental association (EA) tests to search for loci potentially under selection. We further conducted multiple analyses to investigate the effects of environmental variables in driving putatively adaptive genetic variation. Finally, with the aid of the transcriptome sequencing, we annotated a set of functional genes potentially related to environmental adaptation.

## 2 MATERIALS AND METHODS

### 2.1 Sampling and genotyping

**FIGURE 1:**
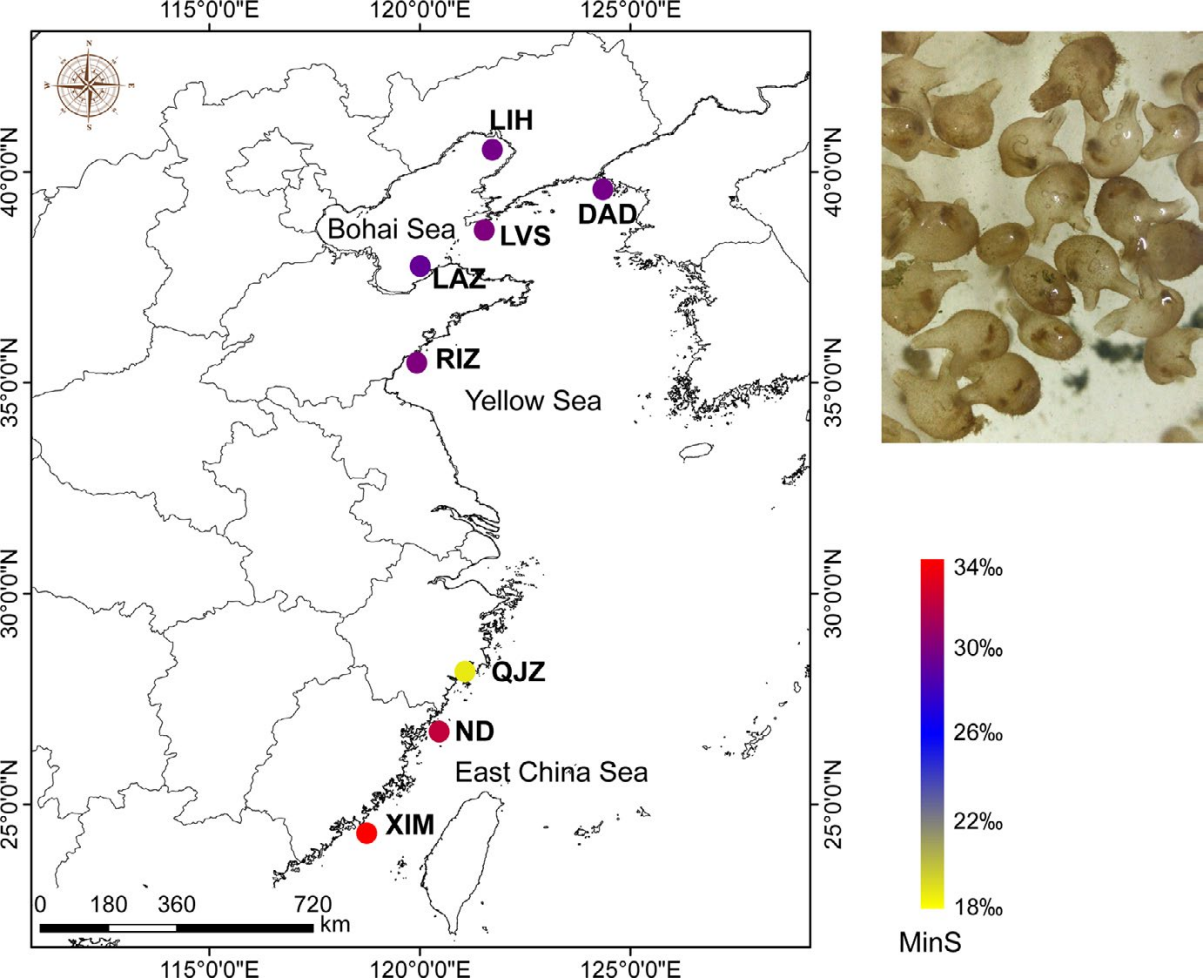
Sampling localities of *Molgula manhattensis* along the Chinese coast. MinS, the lowest monthly average salinity (see more details in Table 1)

A total of 118 M*. manhattensis* individuals were collected from eight sites along the Chinese coast in 2013 (Table 1; Figure 1). Specifically, QJZ was sampled in the Yueqing Bay, which is a typical semi‐enclosed bay with low salinity (approximately 20‰) due to inflows of rivers. The others were collected along the inshore of Chinese coast (Figure 1). Six environmental factors associated with sea surface salinity and temperature (among 1955–2012) were obtained from the World Ocean Atlas 2013 of the National Oceanic and Atmospheric Administration (www.nodc.noaa.gov/OC5/SELECT/woaselect/woaselect.html), including three sea surface salinity matrices: annual average salinity (AveS), the highest monthly average salinity (MaxS), and the lowest monthly average salinity (MinS), and three sea surface temperature matrices: annual average temperature (AveT), the highest monthly average temperature (MaxT), the lowest monthly average temperature (MinT) (Table 1).

**TABLE 1:** Sampling sites information and population genetic variation based on 3,616 putatively neutral single nucleotide polymorphisms (SNPs)

Sampling site	Latitude	Longitude	*N*	T (℃)			S (‰)			*H*o	*H*_E_	*P*_i_	Bottleneck
	(*N*)	(E)		AveT	MaxT	MinT	AveS	MaxS	MinS				
Dandong (DAD)	39.8708	124.3020	21	11.94	24.49	1.02	30.32	31.56	29.72	0.285	0.272	0.279	0.33(0.20)
Linghai (LIH)	40.8837	121.3323	22	12.15	24.43	0.60	30.75	31.64	29.84	0.265	0.268	0.275	0.37(0.16)
Lvshun (LVS)	38.4705	121.1538	10	12.34	24.54	0.89	30.63	31.77	30.00	0.248	0.255	0.269	0.79(0.19)
Laizhou (LAZ)	37.8634	120.3249	12	12.55	24.16	0.44	30.06	31.07	29.12	0.248	0.226	0.238	0.06(0.01)
Rizhao (RIZ)	35.5702	119.6944	15	14.61	25.97	3.38	31.11	31.79	30.07	0.257	0.241	0.251	0.71(0.10)
Qingjiangzhen (QJZ)	28.2891	121.1691	11	17.71	28.49	6.78	24.10	28.42	18.94	0.248	0.232	0.244	0.36(0.11)
Ningde (ND)	26.4819	119.9838	17	22.47	28.36	15.02	32.24	33.87	32.23	0.250	0.259	0.268	0.78(0.15)
Xiamen (XIM)	24.4292	118.1725	10	23.91	28.89	17.55	33.93	34.65	33.04	0.255	0.257	0.272	0.71(0.16)

Abbreviation: *N*, number of individuals; AveT, annual average water temperature; MaxT, the highest monthly average water temperature; MinT, the lowest monthly average water temperature; AveS, annual average water salinity; MaxS, the highest monthly average water salinity; MinS, the lowest monthly average water salinity; *H*_O_, observed heterozygosity; *H*_E_, expected heterozygosity; *P*_i_, nucleotide diversity; Bottleneck, *p*‐values from bottleneck test for excess heterozygosity. *H*_O_, *H*_E_, and *P*_i_ were based on 3,616 putatively neutral SNPs, which were analyzed by bootstrapping 100 random selected neutral SNPs from 3,616 putatively neutral SNPs and then calculated the mean and standard deviation of *p*‐values: mean (stdev) for each population.

Total genomic DNA was extracted using the DNeasy Blood and Tissue Kit (69,506, QIAGEN). The quality and quantity of isolated genomic DNA were examined by a NanoDrop 2000 spectrophotometer (Thermo Fisher Scientific, USA). The 2b‐RAD library construction was conducted as described by Wang et al., (2012) with minor modifications. Briefly, we added NNRW adapters (N refers to any nucleotide, R refers to A or G, W refers to A or T) in order to remove PCR duplicates, which can reduce downstream genotyping errors (Dixon et al., 2015). In order to pool different libraries into a single lane for sequencing, we adopted the multiplexing 2b‐RAD library method by combining 12 barcoded adapters with 24 indexed PCR primers (Guo, Yuan, et al., 2014). For all eight populations, we sequenced three lanes of single‐end sequencing (SE50) on the Illumina Hiseq 4,000 platform (Illumina, USA).

After the high‐throughput sequencing, these libraries were de‐multiplexed based on barcodes and further removed duplicates using a modified perl script (Gao et al., 2018). In addition, the terminal 2 bp nucleotides were removed from each read in order to avoid possible artifacts derived from the ligation sites. After trimming and quality filtering with the “*process_radtags”* module in STACKS v.2.1 (Catchen et al., 2013), we obtained high‐quality reads with the uniform length of 32 bp. Filtered reads were then implemented with STACKS for de novo genotyping due to the lack of a reference genome of *M. manhattensis*. After parameter testing, the optimal parameters set in “*denovo_map.pl”* pipeline program in STACKS were as follows: minimum depth of three to create a stack (*m* = 3) in *ustacks*, maximum number of two mismatches allowed between stacks (*M* = 2) in *ustacks*, two mismatches allowed between sample loci within a catalog (*n* = 2) in *cstacks* and the default for *sstacks* to match stacks of all samples in the population against the catalog (Andrews et al., 2018). The “*populations”* programs in STACKS were used to perform SNP filtering and produce a series of population genetics statistics. To minimize the occurrence of false SNPs, we performed the following strict filtering options as suggested by Rochette and Catchen (2017). Firstly, we discarded RAD tags with stacks depth less than four (*m* = 4), retained bi‐allelic SNP per locus with minor allele frequency (MAF) > 0.05 to reduce false‐positive SNP calls (Rochette & Catchen, 2017). Secondly, we screened for SNPs genotyped in at least 75% of all populations (*p* = 6) and 75% of individuals in a population (*r* = 0.75). Thirdly, we performed the “*write_single_snp”* option with only the first SNP per RAD tag retained in the final set to avoid physical‐linked loci (Andrews et al., 2018). We finally removed SNPs and individuals with more than 20% missing data.

### 2.2 Identifying loci potentially under selection

The main objectives of our study are firstly to provide a dataset of putatively adaptive loci, and then test their relevance to local environmental factors through genotype‐environment matching analysis and RDA analysis, and finally conduct blast alignments to annotate candidate genes under selection. Therefore, our final panel of putatively adaptive loci was composed of loci identified by at least two of three genome scan methods, which can maximize the power of the analysis while reducing the FDR, especially for invasive species with complex demographic histories in different landscapes (Dalongeville et al., 2018; Lotterhos & Whitlock, 2015). Here, we adopted two population differentiation (PD) approaches (BAYESCAN, OUTFLANK) and one environmental association (EA) approach (LFMM) to test for natural selection. Firstly, we identified loci potentially under selection (i.e., outlier SNPs) using a Bayesian approach in BAYESCAN v.2.1 (Foll & Gaggiotii, 2008). The parameter settings of BAYESCAN analysis were as follows to reduce the number of false positives: 20 pilot runs with a length of 5,000 iterations after a burn‐in of 50 000 steps. Candidate outlier loci were defined as those SNPs with false discovery rate (FDR) lower than 5% (*q*‐value < 0.05). Another PD approach, OUTFLANK, which estimated the distribution of *F*_ST_ for neutral loci based on the trimmed distribution of *F*_ST_ values, was adopted to detect *F*_ST_ outliers using the “*OutFLANK*” package in R (Whitlock & Lotterhos, 2015). This method has been reported with a low false discovery rate and a high power in populations with complex demographic histories. We ran OUTFLANK with the following options: RightTrimFraction = 0.05, LeftTrimFraction = 0.05, Hmin = 0.1, q‐threshold = 0.05. As PD analyses just used the “blind” genome scanning without incorporating environmental variation (Rellstab et al., 2015; Villemereuil & Gaggiotti, 2015), we further employed one environmental association (EA) approach, a latent factor mixed‐effect model (LFMM) in the R package “*LEA*” (Frichot & François, 2015). LFMM analysis tests correlate between loci and environmental factors while correcting for unobserved latent factors, which has been shown to be robust in complex models (Caye et al., 2019; Frichot et al., 2013). Firstly, we estimated the number of latent factors using a cross‐entropy criterion with “*snmf*” function in LEA package with 10 repetitions for each *K* value between 1 and 8. Secondly, we performed an environmental association test using the LEA function “*lfmm*” with the best number of latent factor according to “*snmf*” results. In order to reduce the collinearity among environmental variables (Pearson'r test, Table S4), we summarized the environmental variables by running a principal component analysis (PCA) for three temperature variables and three salinity variables, respectively, and retaining only higher order PCs (i.e., PC1‐T, PC1‐S). The “*lfmm*” function was conducted with 100 000 iterations, 10 000 burn‐in cycles and 5 replicates. We then combined *z*‐scores over five runs and used adjusted *p*‐values using the genomic control method implemented in LEA function “*lfmm.pvalues*”, and this recalibration procedure could decrease the false discovery rate in the LFMM test (Frichot & François, 2015). We finally obtained the list of candidate SNPs using the Benjamini–Hochberg procedure with a false discovery rate at 0.05.

### 2.3 Neutral genetic variation and gene flow analyses

Natural selection can potentially influence estimates of neutral genetic diversity and gene flow (Luikart et al., 2003; Waterhouse et al., 2018), and we therefore chose to build a reliable “neutral dataset” by removing all potential outliers obtained from three approaches (BAYESCAN, OUTFLANK and LFMM; see below). We then used PLINK v.1.90 (Purcell et al., 2007) to test for departure from Hardy–Weinberg equilibrium (HWE, *p* <.01) at putatively neutral loci. Genome‐wide population genetic diversity, including observed heterozygosity (*H*o), expected heterozygosity (*H*_E_), and nucleotide diversity (*P*_i_), was calculated with the *Populations* program in STACKS. We further tested whether these populations had experienced recent genetic bottleneck using BOTTLENECK v.1.2.02 (Piry et al., 1999) under the two‐phased mutation model (TPM) with 90% single‐step mutations. Significant heterozygosity excess was evaluated by Wilcoxon signed‐rank test with 1,000 iterations (Yang et al., 2014). These 100 random loci were sampled from the neutral dataset with 1,000 bootstrap iterations.

Population genetic differentiation was examined by calculating the pairwise *F*_ST_ values with 10 000 permutations in ARLEQUIN. The significance level of pairwise *F*_ST_ values was adjusted after sequential Bonferroni correction. Population genetic structure was firstly examined by the discriminant analysis of principal components (DAPC) in the R package “*ADEGENET*” (Jombart, 2008). This multivariate method uses *K*‐means of principal components and Bayesian information criterion (BIC) to infer the best‐supported number of genetic clusters. We used the “*optim.a.score*” function to determine the number of retained principal components (PCs) and discriminant functions (DAs). To complement the DAPC analysis, we also performed one admixture analysis with the Bayesian clustering in STRUCTURE v.2.3 (Pritchard et al., 2000). For the STRUCTURE analysis, we performed 10 independent runs for each *K* (*K* was from one to eight), with 5,000,000 Markov chain Monte Carlo iterations preceded by 500,000 burn‐in steps. The optimal *K* value was identified using the Δ*K* method of Evanno et al., (2005) in STRUCTURE HARVESTER (Earl, 2012), and we used DISTRUCT v.1.1 (Rosenberg, 2004) to visualize population assignments.

We estimated the recent migration rate between populations based on all neutral loci using the modified BayesAss analysis “BA3‐SNPs” (Mussmann et al., 2019). The analysis was run for 10 000 000 iterations after a burn‐in of 1 000 000 steps and sampled every 100th iteration. Mixing parameters for migration rate (m), allele frequency (a), and inbreeding coefficient (f) were adjusted to ensure the final MCMC acceptance rate between 0.35 and 0.45 for each variable (Mussmann et al., 2019). A rough 95% confidence interval (CI) was constructed as (mean ± 1.96 _*_ sdev).

### 2.4 Redundancy analysis and variance partitioning analysis

Redundancy analysis (RDA) and variance partitioning analysis (VPA) were used to investigate the relative contribution of spatial distribution and environmental factors to both putatively neutral and adaptive genetic variation among populations. Because both genetic variation and environmental factors may be spatially autocorrelated (Rellstab et al., 2015), we considered the effect of spatial distribution on population structure using the principal coordinates of neighbor matrices (PCNM) analysis (Benestan et al., 2016; Borcard and Legendre 2002). Spatial factors were assessed by the Euclidian distance matrix with the transformed latitude and longitude of each sampling site with the PCNM method. The PCNM analysis followed the procedure as described in Benestan et al., (2016), using the PCNM package in R. Before the RDA analysis, six environmental factors were firstly standardized using the “*decostand*” function with the standardize method implemented in the R package “*vegan*”. We ran a PCA with “*prcomp*” procedure on six environmental factors to reduce their collinearity. The response variables were characterized with the minor allele frequency (MAF) of putatively adaptive and neutral loci calculated in PLINK v.1.90, respectively. The MAF data were detrended with the Hellinger method implemented in “*vegan*” package in R. We identified significant explanatory variables using “*forward.sel*” function in R. The partial RDA (pRDA) and variance partitioning analysis (VPA) were then used to distinguish the effect of environmental variables from spatial factors. The pRDA analysis was implemented with the R package “*vegan*”. Variance partitioning analysis (VPA) was performed to assess the pure effects of environments and geography as well as their joint effects using the “*varpart*” function with 1,000 permutations.

### 2.5 Transcriptome sequencing and gene annotation

Due to the limited genomic resources available for *M. manhattensis*, we used the transcriptome of this species as a reference to annotate outlier loci, which was a cost‐effective method for nonmodel species without reference genomes (Benestan et al., 2016; Lamichhaney et al., 2012; Thomas et al., 2017). Firstly, we conducted transcriptome sequencing of three *M. manhattensis* individuals, and transcripts were performed with de novo assembly with Trinity v.2.4.0 (Grabherr et al., 2011). Open reading frame (ORF) over 100 amino acids in length was firstly extracted from the Trinity assembly to identify candidate protein‐coding regions by the program TransDecoder in Trinity. The transcripts with a significant coding potential were annotated according to multiple databases, for example, GenBank's NR protein database, UniProt's Swiss‐Prot database, and PFAM database, respectively, using Trinotate v.3.0.2 with an e‐value cutoff 1.00E‐20 to create the functional gene database of *M. manhattensis* (unpublished results). The RAD tags containing candidate adaptive loci were blasted against the complete transcriptome of *M. manhattensis* using BLAST v.2.5.0+ (McGinnis and Madden 2004) with an e‐value threshold of 1.00E‐05 and a maximum of two nucleotide mismatches. A set of functionally genes were yielded, and their functions were assessed by GO annotation terms. To further characterize whether these SNPs were synonymous or nonsynonymous changes, we firstly translated contig sequences containing candidate loci into codons in the six reading frames with the program BIOEDIT v.7.1 and subsequently translated the codon containing the SNP into an amino acid based on the location of the start codon (Benestan et al., 2016).

## 3 RESULTS

### 3.1 SNP genotyping

After raw data processing, a total of 769 983 RAD tags were obtained from three lanes of Illumina high‐throughput sequencing (Table S1). The depth of RAD tags for each sample varied from 10 to 22 x. After the strict SNP filtering steps in STACKS, we finally obtained a panel of 6,635 high‐quality genome‐wide SNPs for subsequent analyses (Table S1).

### 3.2 Neutral genetic variation and gene flow

**FIGURE 2:**
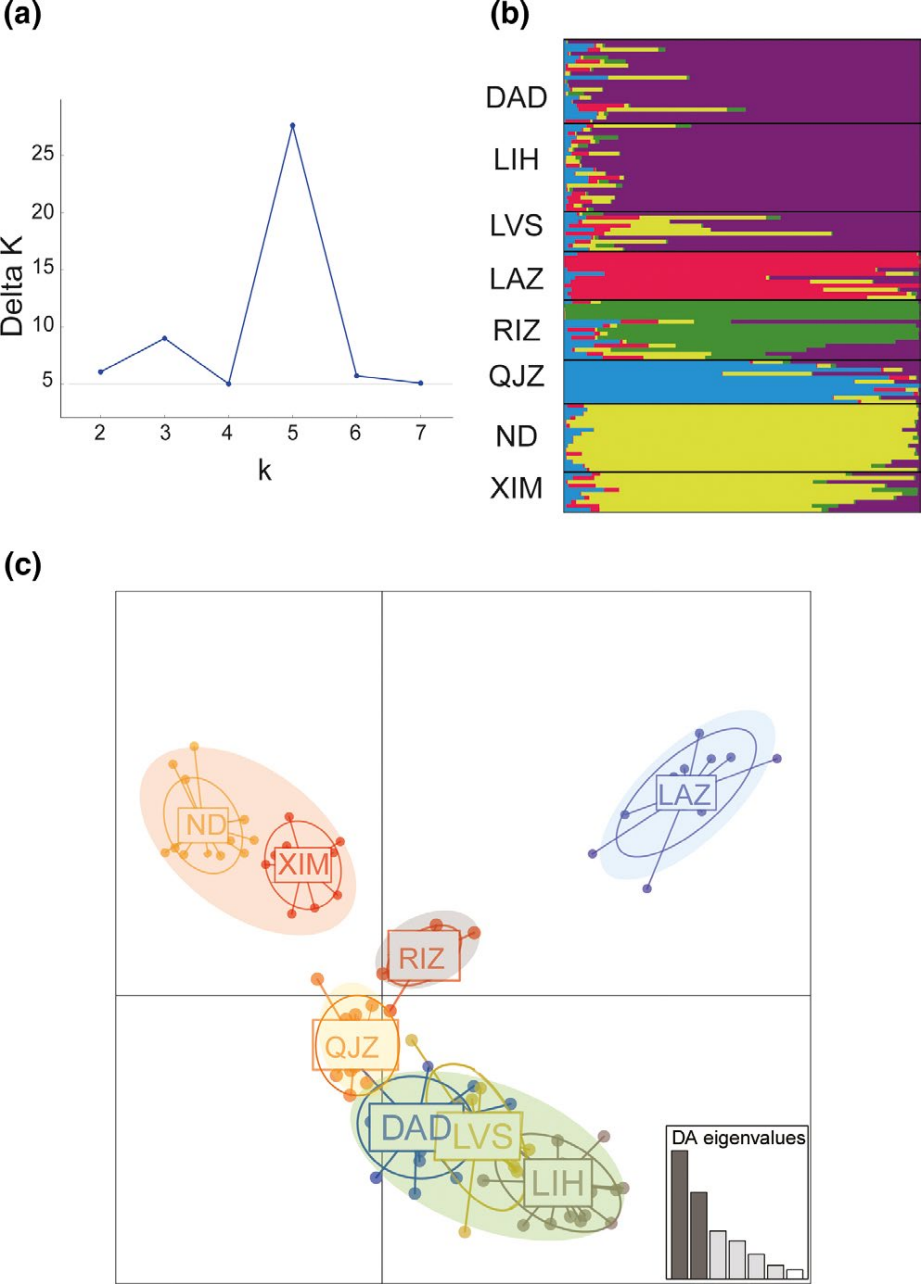
Population assignment in eight populations of *Molgula manhattensis* using the whole 6,635 SNPs. (a) The optimal *K* value using the Δ*K* method in STRUCTURE HARVESTER; (b) STRUCTURE results at *K* = 5; (c) Discriminant analysis of principal components (DAPC) plot

After removing all potential non‐neutral SNPs (i.e., a total of 2,335 loci based on three genome scan methods), 3,616 candidate neutral SNPs with Hardy–Weinberg equilibrium were finally retained, constituting a rather reliable and conservative panel for subsequent analyses of neutral genetic patterns. Based on 3,616 neutral SNPs, we revealed a similar level of genetic diversity across all eight sampling sites. The observed heterozygosity (*H*o) ranged from 0.248 to 0.285, expected heterozygosity (*H*_E_) varied from 0.226 to 0.272, and nucleotide diversity (*P*_i_) ranged from 0.238 to 0.279 (Table 1). The Wilcoxon sign rank test for excess heterozygosity revealed no sign of significant population bottleneck (Table 1). Most populations exhibited significant genetic differentiation (except RIZ with five nonsignificant pairwise *F*_ST_ values; Table S2). For the whole SNP dataset, eight populations were assigned into five genetic clusters (optimal *K* = 5; Figure 2a) according to the Bayesian assignment using STRUCTURE. The first cluster including three sampling sites DAD, LIH, and LVS; ND and XIM were assigned into the second cluster; and the other three sites, LAZ, RIZ, and QJZ, were assigned to three different clusters (Figure 2b). The DAPC analysis with the optimal number of PCs = 40 and DAs = 6 also showed the same pattern of five groups as in STRUCTURE (Figure 2c). In addition, for the DAPC analysis with neutral dataset, four clusters were revealed according to the lowest BIC value (*k* = 4): one cluster with four populations (LIH, DAD, LVS, and RIZ), one cluster with two populations (ND and XIM), and the other two clusters with two different populations LAZ and QJZ (Figure S1a), which was consistent with the *F*st results. BayesAss analysis revealed extremely low recent migrant rates (Table S3).

### 3.3 Candidate adaptive loci detection

**FIGURE 3:**
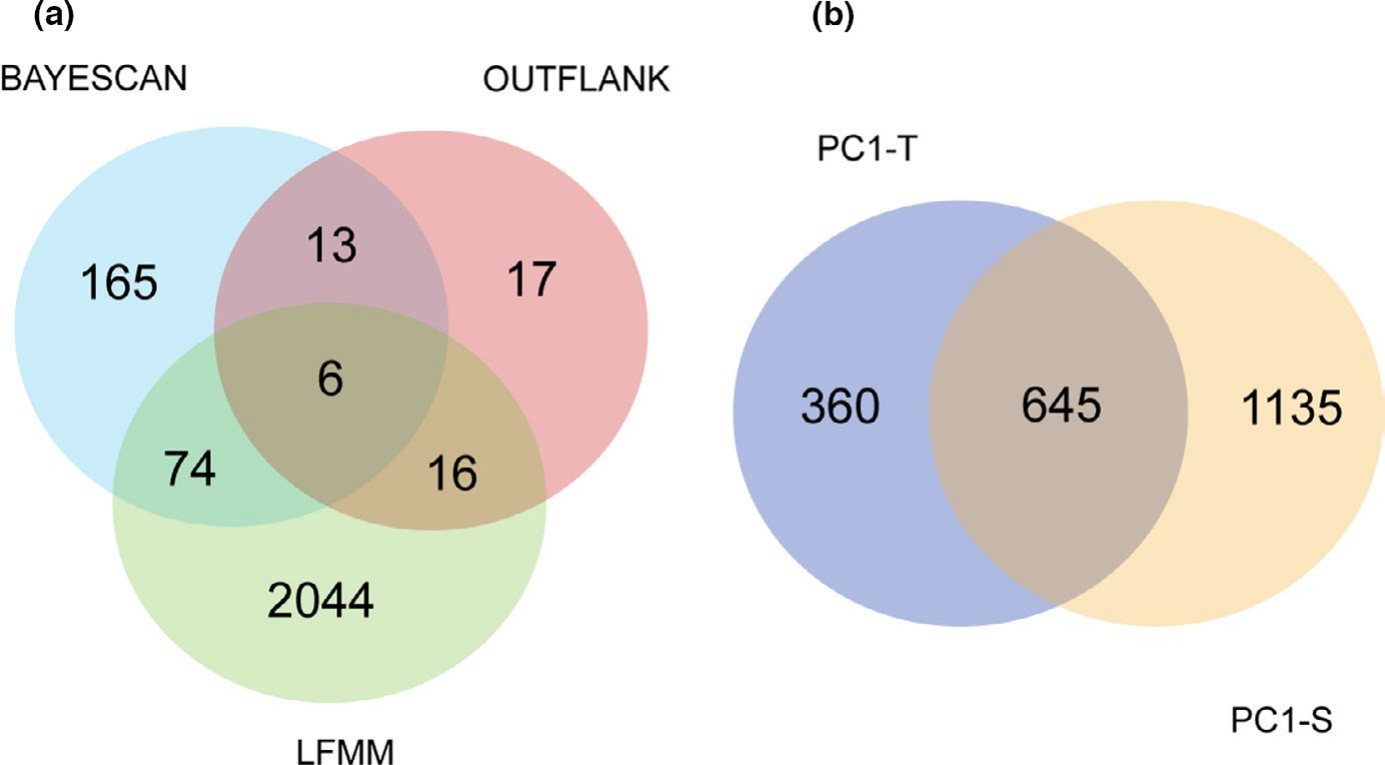
Candidate adaptive loci detection. (a) The number of loci identified by BAYESCAN, OUTFLANK, and LFMM analyses; (b) the number of loci significantly correlated with temperature‐related variables (PC1‐T) and salinity‐related variables (PC1‐S) in LFMM. PC1‐T: the first axis of a principal component analysis for three temperature variables; PC1‐S: the first axis of a principal component analysis for three salinity variables

For the BAYESCAN and OUTFLANK analyses, we identified 258 and 52 loci, respectively (Figure 3a). For the LFMM, the best number of latent factors was set to five with the minimal cross‐entropy according to “*snmf*” results (Figure S2), which was also consistent with the DAPC and STRUCTURE results (Figure 2a). The first axis of PCA for temperature‐related variables (PC1‐T) and salinity‐related variables (PC1‐S) both explained over 96% of the total variation (PC1‐T: 96.96%; PC1‐S: 96.22%). After a recalibration procedure with the genomic control method, we finally obtained a total of 2,140 loci, including 1,005 associated with PC1‐T and 1,780 associated with PC1‐S (Figure 3b). Interestingly, 645 SNPs (30.14%) were shared among these two groups of environmental variables, whereas the others were group‐specific, including 360 (16.82%) temperature‐specific loci and 1,135 (53.04%) salinity‐specific loci. Collectively, 109 SNPs identified by at least two of three genome scan methods were considered as putatively adaptive loci for subsequent analyses. We found that the distribution of allele frequency at putatively adaptive loci largely varied among populations. Based on the allele frequency of 109 candidate loci, eight populations were divided into five groups (Figure S3), which was consistent with the genetic patterns based on putatively adaptive SNPs as revealed by the DAPC analysis (Figure S1b). Several loci, such as 8700_26, 32625_5, 17568_3, 18471_28, exhibited higher allele frequencies in the two south populations (ND and XIM) when compared to the others (Figure S3). For another south population QJZ, allele frequency at some loci, such as 31311_0, 30284_22, 4585_28, was the highest (allele frequency > 0.8), whereas a large number of loci (79 out of 109) had an allele frequency of zero (Figure S3).

### 3.4 The effect of environmental variables in driving genetic variation

**FIGURE 4:**
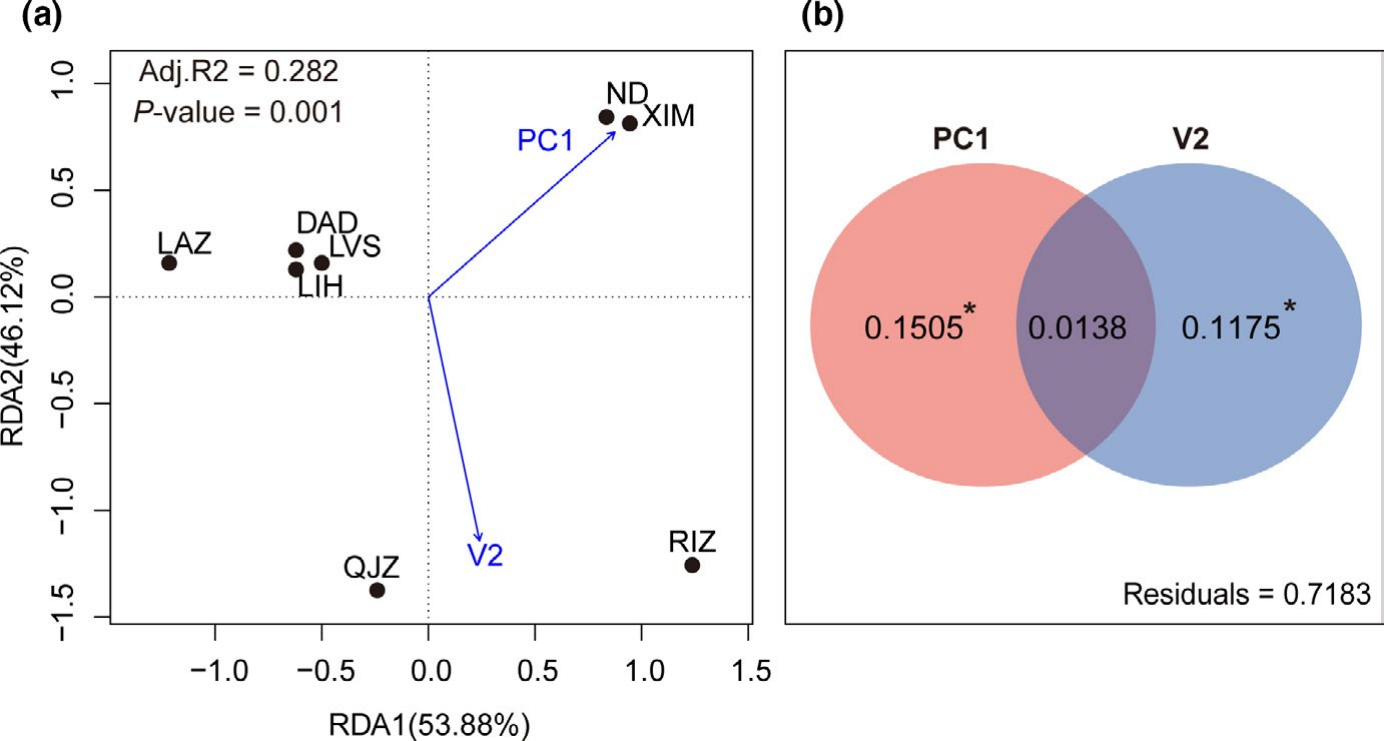
Redundancy analysis and variance partitioning analysis. (a) Redundancy analysis (RDA) performed on 109 candidate adaptive loci; (b) results of variance partitioning analysis (VPA) performed to assess the relative role of environmental and spatial variables in shaping putatively adaptive genetic variation. PC1: the first axis of a principal component analysis for six environmental variables; V2: one spatial vector obtained from the PCNM analysis; *: *p* <.05

According to the principal coordinates of neighbor matrices (PCNM) analysis, two vectors (V1 and V2) were retained as representative spatial distribution variables. Four variables, including the first two principal components retained by PCA analysis of environmental factors (the proportion explained by PC1 and PC2: 62.01%, 37.55%) and two spatial vectors (V1 and V2), were firstly considered as the primary explanatory variables in the RDA model. PC1 and V2 were then selected as the significant predictors of the putatively adaptive genetic variation according to the forward selection (*p‐*PC1 = 0.012; *p‐*V2 = 0.013) in the RDA analysis. This parsimonious RDA model was significant (*P*‐model = 0.001) with an adjusted coefficient of determination (Adj.R^2^) of 0.282 (Table 2; Figure 4a). When partitioning the relative effect of environmental variables and spatial distribution on putatively adaptive genetic variation, the variation purely explained by environments was higher (15.05%) than that explained by spatial distribution (11.75%) (Table 2; Figure 4b). We also assessed the relative contribution of environmental and spatial distribution variables in influencing population genetic variation for the putatively neutral loci; however, we did not detect the significance among eight variables according to the forward selection procedure in RDA analysis. In addition, we assessed the role of sea surface temperature and salinity (PC1‐T, PC1‐S; see details in LFMM analysis) in driving putatively adaptive genetic variation, and only PC1‐S was significantly selected by the forward selection procedure (*p‐*PC1‐S = 0.039). Moreover, salinity‐related variables (PC1‐S) provided a higher proportion than temperature‐related variables (PC1‐T) in explaining putatively adaptive genetic variation (10.98% versus 6.98%; Table 2).

**TABLE 2:** Redundancy analysis (RDA) and partial RDA (pRDA) results

Analyses	Selected variables (forward selection)	Adj.R^2^	*P*‐model
RDA	PC1*	0.2817	***
	PC2		
	V1		
	V2*		
pRDA	PC1	0.1505	*
	V2	0.1175	*
RDA	PC1‐T	0.1640	*
	PC1‐S*		
pRDA	PC1‐T	0.0698	0.1380
	PC1‐S	0.1098	0.2650

Note

Significant values (*P*‐values) were indicated with the following symbols: *: 0.05; ***: 0.001.

### 3.5 Gene annotation of candidate adaptive loci

Based on 109 candidate adaptive loci, 28 RAD tags containing candidate adaptive loci were unambiguously matched to the complete transcriptome of *M. manhattensis*. A total of 14 functional genes were successfully annotated according to our gene database of *M. manhattensis,* and seven carried nonsynonymous substitutions leading to amino acid changes (Table 3). For instance, two nonsynonymous SNPs, 8700_26 (T/C) and 32625_5 (A/G), lead to amino acid change from phenylalanine (Phe) to leucine (Leu) and from lysine (Lys) to glutamic acid (Glu), respectively. Both are located in the integrin alpha‐L (ITGAL) gene, and this gene can participate in apoptotic process, metal ion binding and cadherin binding based on GO annotation (Table 3). Another nonsynonymous SNP 19292_8 (G/A, Glu/Lys) is located in the titin (TTN) gene, which plays a role in the assembly and functioning of muscles as well as chromosome condensation and segregation during mitosis (Table 3). Among these candidate SNPs with synonymous substitutions, the SNP 8499_18 is located in the solute carrier family 9 member C1 gene (SLC9C1, also called sodium‐hydrogen exchanger 10 gene), which is a member of the Na^+^/H^+^ exchanger family involved in the electroneutral exchange of Na^+^ and H^+^. Another SNP 37385_3 was also located in one gene of the solute carrier (SLC) subfamily, the solute carrier family 5 member 1 (SLC5A1). This gene can get involved in sodium ion transport, glucose transmembrane transport, and intestinal hexose absorption (Table 3).

**TABLE 3:** Functional annotation of genes derived from putatively adaptive SNPs against the *Molgula manhattensis* transcriptome

SNP	Gene_ID	Amino acid change	Protein names	Gene Names	GO annotation	E‐value
1838_8	TRINITY_DN24651_c0_g2	CCG/TCG = Pro/Ser	protein CBFA2T1	RUNX1T1	DNA‐binding transcription factor activity,	6.05E−09
					metal ion binding,	
					transcription corepressor activity,	
					generation of precursor metabolites and energy,	
					negative regulation of fat cell differentiation	
4535_14	TRINITY_DN27611_c1_g1	ATA/ATC = Ile/Ile	multidrug resistance protein 1	MDR1	cellular response to oxidative stress,	5.00E−10
					cellular response to drug,	
					pathogenesis,	
					drug transmembrane transport	
8499_18	TRINITY_DN15956_c0_g2	GAC/GAT = Asp/Asp	solute carrier family 9 member C1	SLC9C1	ion channel activity,	5.00E−10
					potassium: proton antiporter activity,	
					sodium: proton antiporter activity,	
					regulation of intracellular pH	
11269_27	TRINITY_DN19778_c3_g3	GCA/GCG = Ala/Ala	glutamate receptor ionotropic, kainate 2	GRIK2	ubiquitin protein ligase binding,	4.68E−10
					cellular calcium ion homeostasis,	
					extracellularly glutamate‐gated ion channel activity,	
					kainate selective glutamate receptor activity	
17358_6	TRINITY_DN23248_c0_g1	GAC/GAT = Asp/Asp	DNA repair protein RAD50	RAD50	DNA repair,	4.68E−10
					metal ion binding,	
					telomere maintenance,	
					regulation of mitotic recombination	
19292_8	TRINITY_DN27897_c22_g1	GAG/AAG = Glu/Lys	titin	TTN	response to calcium ion,	4.68E−10
					mitotic chromosome condensation,	
					muscle contraction,	
					protein kinase A signaling	
20518_17	TRINITY_DN25991_c1_g1	ATA/ATC = Ile/Ile	phosphatidylserine decarboxylase proenzyme	PSD1	phosphatidylethanolamine biosynthetic process	4.68E−10
21411_31	TRINITY_DN18963_c0_g1	CTG/CCG = Leu/Pro	tetratricopeptide repeat protein 36	TTC36	determination of heart left/right asymmetry,	2.00E−09
					otolith morphogenesis,	
					cilium assembly	
25823_14	TRINITY_DN24740_c0_g1	AGT/AGA = Ser/Arg	prolactin regulatory element‐binding protein‐like	PREB	GTPase activator activity,	4.68E−10
					protein secretion,	
					protein processing in endoplasmic reticulum	
29286_22	TRINITY_DN23730_c1_g1	TCA/TCG = Ser/Ser	Heparan sulfate 2‐O‐sulfotransferase 1	HS2ST1	glycosaminoglycan biosynthetic process,	4.68E−10
					Golgi membrane,	
					integral component of membrane	
33310_16	TRINITY_DN13671_c0_g1	GGA/GAA = Gly/Glu	deoxycytidine kinase	DCK	deoxycytidine kinase activity,	5E−10
					ATP binding, kinase activity,	
					transferase activity	
37385_3	TRINITY_DN25048_c0_g1	GCT/GCA = Ala/Ala	solute carrier family 5 member 1	SLC5A1	glucose: sodium symporter activity,	5E−10
					glucose transmembrane transport,	
					sodium ion transport,	
					intestinal hexose absorption	
4585_28	TRINITY_DN17675_c0_g1	CTG/CCG = Leu/Pro	aldehyde dehydrogenase 2 family	ALDH2	sphingolipid biosynthetic process,	2.00E−08
					catabolic process,	
					response to hyperoxia,	
					response to lipopolysaccharide	
26609_3	TRINITY_DN17675_c0_g1	GCC/GCT = Ala/Ala	aldehyde dehydrogenase 2 family	ALDH2	sphingolipid biosynthetic process,	2.00E−08
					catabolic process	
					response to hyperoxia,	
					response to lipopolysaccharide	
8700_26	TRINITY_DN24977_c0_g1	TTC/CTC = Phe/Leu	integrin alpha‐L	ITGAL	positive regulation of apoptotic process,	4.68E−10
					cadherin binding,	
					metal ion binding,	
					regulation of immune response,	
					ectodermal cell differentiation,	
					integrin‐mediated signaling pathway	
32625_5	TRINITY_DN24977_c0_g1	AAG/GAG = Lys/Glu	integrin alpha‐L	ITGAL	positive regulation of apoptotic process,	4.68E−10
					cadherin binding,	
					metal ion binding,	
					regulation of immune response,	
					ectodermal cell differentiation,	
					integrin‐mediated signaling pathway	

Note

These 14 genes were annotated from 109 candidate adaptive SNPs. Gene annotation was performed with local BLAST of candidate sequences against the complete transcriptome of *Molgula manhattensis*.

## 4 DISCUSSION

In this study, we employed genome‐wide SNPs to assess population genetic diversity and gene flow and then investigate genomic signatures of environmental adaptation in a marine invasive ascidian, *Molgula manhattensis*, along the Chinese coast. Multiple analyses illustrated that environmental factors, especially salinity‐related variables, might represent strong evolutionary forces in driving putatively adaptive genetic divergence. Candidate adaptive loci were successfully annotated to a group of functional genes with potential roles in contributing to environmental adaptations. These genes are good candidates for further studies on the evolutionary dynamics of environmental adaptations in marine invasive species.

### 4.1 Environment‐driven adaptive genetic divergence

Dissecting the effect of environmental conditions on population genetic variation across introduced populations would provide us a better understanding on how invasive species respond to different environments during range expansions (Papacostas et al., 2017; White et al., 2013). For newly introduced populations, demographic processes, such as genetic drift and gene flow, might confound the effect of environmental factors when investigating signals of natural selection (Ahrens et al., 2018; Lotterhos & Whitlock, 2014). In our study, neutral genetic variation was first and foremost analyzed, as it is the null hypothesis of statistical tests for selection. Based on a rather reliable panel of 3,616 neutral SNPs, all eight sampling sites were detected with a similar level of population genetic diversity and no signal of significant population bottleneck, indicating that these populations were less likelihood to experience strong genetic drift. These findings agree with previous results of *Bottleneck* analysis using 12 microsatellite markers in *M. manhattensis* populations (Chen, Li, et al., 2017). Gene flow could play a role in influencing population divergence and may weaken, or even completely eliminate, signatures of local adaptation (Lenormand, 2002). We detected low recent migration rates, which is consistent with the observed significant genetic divergence among most populations. The observed population genetic heterogeneity at large geographical scales and low gene flow among distant populations were in contrast to the findings in Chen, Li, et al., (2017), which uncovered low population differentiation and high genetic similarity among several geographical‐distant sites (i.e., DAD versus ND). This discordance of population genetic pattern may result from the adopted molecular makers among these two studies. Compared with population genetic studies with a limited number of genetic makers, genome‐wide SNPs here could contribute to the detection of subtle genetic structure and accurate estimation of gene flow (Benestan et al., 2015; Blanco‐Bercial & Bucklin, 2016).

*M. manhattensis* has limited natural dispersal ability, mainly owing to a short pelagic larval phrase, sessile adults, and a high self‐recruitment rate (Chen, Li, et al., 2017). Considering that *M. manhattensis* is commonly found in the aquaculture ponds and facilities with a high density (Zheng, 1995), human‐mediated dispersal *via* frequent aquaculture transfers might exist as suggested by the previous study (Chen, Li, et al., 2017) and studies with other invasive ascidians (i.e., *Ciona intestinalis* in Zhan et al., 2012; *Botrylloides violaceus* in Bock et al., 2011; *Botryllus schlosseri* in Bock et al., 2012). Therefore, the significant genotype‐environment matching pattern in this study suggests that the underlying gene flow among populations is low enough to allow for local adaptation.

In our study, we revealed a large number of candidate environment‐related loci (i.e., 2,140 in LFMM) and the key role of environmental factors in affecting putatively adaptive genetic variation. Similar genotype–environment matching patterns have been recently uncovered in a variety of marine organisms, including the American lobster (Benestan et al., 2016), Chinese sea bass (Zhao et al., 2018), and greenlip abalone (Sandoval‐Castillo et al., 2018). For example, Sandoval‐Castillo et al., (2018) detected population adaptive divergence in *Haliotis laevigata* along the coast in southern Australia and the observed adaptive variation was significantly correlated with the minimum sea surface temperature and oxygen concentration. Natural selection derived from differed environments can contribute to the genotype–environment matching pattern with an indication of local adaptation (Rellstab et al., 2015; Villemereuil & Gaggiotti, 2015). However, for invasive species, both adaptation occurring before introduction in the native range (pre‐introduction adaptation) and after introduction in the invasive range (postintroduction adaptation) may create the observed environment‐associated genetic variation in recently introduced populations (Bock et al., 2015; Colautti & Lau, 2015). Previous studies suggested that local adaptation and invasion success would be more likely to occur with pre‐adapted genotypes if there was a close match between source and recipient environments, such as anthropogenically induced adaptation to invade (AIAI) (Hufbauer et al., 2012). However, such parallel introduction alone is less likely to establish the obvious genotype–environment matching pattern and local adaptation in diverse introduced regions in our study (Colautti & Lau, 2015). In addition, postintroduction evolution, that is, contemporary evolution, can urge non‐native species to rapidly respond to different environments and increase invasion success (Bock et al., 2015; Prentis et al., 2008). For example, Chen et al., (2018) detected rapid evolution after a recent introduction of *C. robusta* to harsh environments in the Red Sea. Therefore, further efforts by sampling the source regions of *M. manhattensis* are supposed to be conducted to distinguish the relative contribution of these two categories of adaptation in driving the observed genotype‐environment matching pattern (Colautti & Lau, 2015).

Our sampling range spans more than 15 degrees of latitude along the Chinese coast and the sampling sites are characterized with contrasting environmental conditions, such as salinity and temperature. Multiple lines of evidence in our study suggest that environmental conditions, especially salinity‐related variables, significantly contribute to the adaptive genetic variation among populations. Our RDA results revealed that environmental variables accounted for more in explaining adaptive genetic variation when compared with spatial distribution (Table 2; Figure 4b). Furthermore, salinity‐related variables were significantly selected and provided a higher proportion than temperature‐related ones (Table 2). Compared with temperature‐related variables, a larger number of candidate loci was also significantly associated with salinity (PC1‐S), most of which belonged to the salinity‐specific (Figure 2b). As shown in the variance partitioning analysis, there was a large proportion unexplained by the environmental variables (PC1) and spatial variables (V2), suggesting that many other biotic and abiotic variables, such as dissolved oxygen, pH, various pollutants, and biological interactions, may also affect the observed adaptive genetic variation. Seawater salinity stressors, that is, high and low salinity, can both directly and indirectly influence various aspects of marine organisms, such as reproduction, osmoregulatory, metabolic processes, and immune system response (Renborg et al., 2014). Empirical evidence revealed the link between physiological and metabolic traits with salinity challenges in *M. manhattensis*. For example, Cao et al., (2012) documented that oxygen consumption was significantly correlated with salinity conditions in *M. manhattensis* and a low value was detected at high salinity. Salinity stressors can also largely reduce the ingestion rate of this species (Cao et al., 2012). In addition, different salinity conditions have an effect on the developmental process of tunicates, such as *C. intestinalis*, especially for early life stages including embryos and larvae (Renborg et al., 2014). Likewise, changes of DNA methylation profiles associated with responses to different salinity have been evidenced in other invasive ascidians (e.g., Huang et al., 2017; Ni et al., 2018).

### 4.2 Candidate key genes potentially involved in environmental adaptations

Uncovering candidate genes potentially for adaptation would provide direct evidence for genetic basis of adaptive evolution in response to changing environments (Lin et al., 2017). Given the short length of tags generated by the RADseq approach and limited genomic resources of nonmodel species, the use of transcriptome as a reference has been demonstrated to be a credible and cost‐effective way to annotate outlier loci (Ahrens et al., 2018; Benestan et al., 2016; Christmas et al., 2016; Lamichhaney et al., 2012). However, those loci located in noncoding regions could fail to get annotated but may be important to selection. For example, candidate variants in gene promoter regions would regulate gene expression that influence phenotypic variation (Grubert et al., 2015). In addition, the RADseq approach usually only collects a fraction of the genome and we may miss those loci or genes that are not closely linked to these identified SNPs in the genome (Lowry et al., 2017; Hoban et al., 2016). These technical issues are major obstacles when using the RADseq and transcriptome approaches to investigate the genomic basis of local adaptation. In order to avoid false positives in this study, we used extremely strict criteria to identify 14 protein‐coding genes with nonsynonymous/synonymous polymorphisms, and these genes are candidates for further detailed investigation of salinity adaptation mechanisms in marine invasive species.

Two candidate synonymous SNPs 37385_3 and 8499_18 are located in the SLC5A1 and SLC9C1 genes, respectively, both belonging to the solute carrier (SLC) gene superfamily. Adaptive response to salinity stressors of marine species requires the effective ion transport and osmoregulatory processes (Fiol & Kültz, 2007). The SLC5A1 gene is a member of the Na^+^/glucose cotransporter gene family, which can mediate the transport of glucose and structurally related substances across cellular membranes by Na^+ ^cotransport. This gene was reported to get involved in salinity adaptation and osmoregulation in the liver of spotted sea bass (Zhang et al., 2017). Another candidate gene, the sodium/hydrogen exchanger 1 (SLC9C1), encodes Na^+^/H^+^ exchanger (NHE) protein to regulate intracellular pH and acid–base homeostasis. Multiple studies also indicated the crucial role of sodium/hydrogen exchanger genes in osmoregulation in a variety of teleost species (Verri et al., 2012). In addition, we also obtained several functional genes associated with immune responses to cope with environmental stressors, such as the integrin alpha‐L (ITGAL), multidrug resistance protein 1 (MDR1), and DNA repair protein RAD50 (RAD50). Environmental pressures such as temperature and salinity challenges can make organisms more vulnerable to pathogen infections and diseases, and these tunicates reply on the innate immunity to cope with these challenges (Shida et al., 2003). For instance, the integrin alpha‐L (ITGAL) has been reported as a receptor of the complement component 3 (C3)‐like molecule in the complement system during innate immune response of *Ciona intestinalis* (Shida et al., 2003) and *Halocynthia roretzi* (Miyazawa et al., 2001). Further research should be conducted to confirm their adaptive significance and functional mechanisms in salinity adaptation by using gene expression analyses or directly performing genetic manipulation (i.e., RNA interference, CRISPR/Cas9 gene) after challenge experiments.

## 5 CONCLUSION

Utilizing a wide geographical coverage for *M. manhattensis* populations along the Chinese coast, we found that local environmental factors, that is, salinity‐related variables, might drive putatively adaptive genetic variation among varied invasive populations. Furthermore, we identified a set of putatively adaptive loci and functional genes, which are good candidates in studying the evolutionary dynamics of environmental adaptations in marine species. Collectively, our findings confirm the hypothesis that differed local environments could drive adaptive genetic divergence among populations and leave detectable signatures in the genome of marine invaders. Our results suggest that local environment‐driven adaptation might contribute to range expansions and invasion success into varied environments. We further highlight the necessity to incorporate local adaptation dynamics into the prevention and control strategies of biological invasions.

## CONFLICT OF INTEREST

The authors declare no conflict of interest.

## AUTHOR CONTRIBUTIONS

**Yiyong Chen:** Data curation (lead); Formal analysis (lead); Methodology (lead); Software (lead); Validation (lead); Visualization (lead); Writing‐original draft (lead); Writing‐review & editing (lead). **Yangchun Gao:** Formal analysis (equal); Methodology (equal); Software (equal); Validation (equal); Visualization (equal); Writing‐original draft (equal); Writing‐review & editing (equal). **Xuena Huang:** Data curation (equal); Formal analysis (equal); Investigation (equal); Methodology (equal); Software (equal); Validation (equal); Visualization (equal); Writing‐original draft (equal); Writing‐review & editing (equal). **Shiguo Li:** Data curation (equal); Formal analysis (equal); Investigation (equal); Methodology (equal); Software (equal); Validation (equal); Visualization (equal); Writing‐original draft (equal); Writing‐review & editing (equal). **Aibin Zhan:** Conceptualization (lead); Formal analysis (equal); Funding acquisition (lead); Investigation (lead); Methodology (equal); Project administration (lead); Resources (lead); Software (equal); Supervision (lead); Validation (equal); Visualization (equal); Writing‐original draft (lead); Writing‐review & editing (lead).

## Supporting information

Supplementary Material

## Data Availability

Raw data of 2b‐RAD sequencing for *Molgula manhattensis* used in this study have been deposited into Dryad (https://doi.org/10.5061/dryad.18931zcwf).
