# Supplementary material for: Local environment‐driven adaptive evolution in a marine invasive ascidian (Molgula manhattensis)
Source: Ecol Evol. 2021 Mar 6;11(9):4252–66. doi: 10.1002/ece3.7322 (PMC8093682; doi:10.1002/ece3.7322)

**Table S1.** Summary statistics on high-throughput sequencing data for each filtering step.

| Filtering steps | Number |
| --- | --- |
| total clean reads | 545,537,221 |
| total RAD tags | 796,983 |
| SNP filters： | 6,635 |
| *m* >= 4x |
| *H*_O_ < 0.5 |
| MAF > 0.05 |
| *r* = 0.75 |
| *p* = 6 |

† *m*: stacks depth; *H*o: maximum observed heterozygosity; MAF: minimum minor allele frequency; *r*: minimum percentage of individuals in a population required to process a locus for that population; *p*: minimum number of populations a locus must be present in to process a locus.

**Table S2.** Estimates of population genetic differentiation based on the whole neutral dataset (pairwise *F*_ST_, below diagonal) for the *Molgula manhattensis* across eight populations in China.

| Population | DAD | LIH | LVS | LAZ | RIZ | QJZ | ND | XIM |
| --- | --- | --- | --- | --- | --- | --- | --- | --- |
| DAD | **** |  |  |  |  |  |  |  |
| LIH | **0.020** | **** |  |  |  |  |  |  |
| LVS | **0.012** | **0.006** | **** |  |  |  |  |  |
| LAZ | **0.030** | **0.025** | **0.020** | **** |  |  |  |  |
| RIZ | -0.020 | -0.027 | -0.028 | **0.012** | **** |  |  |  |
| QJZ | **0.052** | **0.050** | **0.044** | **0.068** | **0.029** | **** |  |  |
| ND | **0.031** | **0.028** | **0.017** | **0.043** | -0.012 | **0.059** | **** |  |
| XIM | **0.025** | **0.022** | **0.013** | **0.039** | -0.017 | **0.054** | **0.012** | **** |

† Bold numbers indicate statistical significance after sequential Bonferroni correction.

**Table S3.** Results of recent migration using BayesAss3-SNPs based on the whole neutral dataset.

† Recipient and source localities are given in rows and columns, respectively (e.g. LIH is estimated to have 0.017 proportion of recent migrant ancestry from DAD). Values across the diagonal refer to the proportion of non-migrant ancestry in each population and those in bold are with 95% confidence intervals (CIs) not including zero.

|  | DAD | LIH | LVS | LAZ | RIZ | QJZ | ND | XIM |
| --- | --- | --- | --- | --- | --- | --- | --- | --- |
| DAD | **0.886** | 0.046 | 0.011 | 0.012 | 0.012 | 0.011 | 0.011 | 0.012 |
| LIH | 0.017 | **0.917** | 0.011 | 0.011 | 0.011 | 0.011 | 0.011 | 0.011 |
| LVS | 0.018 | 0.029 | **0.860** | 0.019 | 0.019 | 0.019 | 0.018 | 0.019 |
| LAZ | 0.016 | 0.017 | 0.017 | **0.883** | 0.017 | 0.017 | 0.017 | 0.017 |
| RIZ | 0.015 | 0.015 | 0.015 | 0.014 | **0.898** | 0.015 | 0.015 | 0.014 |
| QJZ | 0.017 | 0.033 | 0.018 | 0.017 | 0.017 | **0.862** | 0.018 | 0.018 |
| ND | 0.014 | 0.014 | 0.013 | 0.013 | 0.013 | 0.013 | **0.907** | 0.013 |
| XIM | 0.018 | 0.023 | 0.018 | 0.018 | 0.019 | 0.018 | 0.109 | **0.776** |

**Table S4.** Pearson’ r test of six environmental variables.

|  | AveT | MaxT | MinT | AveS | MaxS | MinS |
| --- | --- | --- | --- | --- | --- | --- |
| AveT | —— | 0.006 | 0 | 0.560 | 0.288 | 0.723 |
| MaxT | 0.938** | —— | 0.015 | 0.933 | 0.711 | 0.754 |
| MinT | 0.994** | 0.899* | —— | 0.438 | 0.204 | 0.587 |
| AveS | 0.302 | -0.045 | 0.395 | —— | 0.001 | 0 |
| MaxS | 0.523 | 0.195 | 0.604 | 0.969** | —— | 0.006 |
| MinS | 0.187 | -0.165 | 0.283 | 0.987** | 0.934** | —— |

† Values below the diagonal are the correlation coefficients (*r)* in the Pearson’ r test; Values above the diagonal are the significance (*p)* in the Pearson’ r test. *p* < 0.05; **：*p* < 0.01.

**Figure S1.** Discriminant analysis of principal components (DAPC) plots with (a) 3616 putatively neutral SNPs and (b) 109 putatively adaptive SNPs.

**Figure S2.** Values of the cross-entropy criterion as a function of the number of factors in the “*snmf*” function in LEA package.

**Figure S3.** Heat map of allele frequency of 109 candidate adaptive loci.

† Rows represent different populations and columns represent specific loci. Colours represent minor allele frequencies (MAF).

**Figure S4.** The environmental PCA analysis based on six environmental variables.


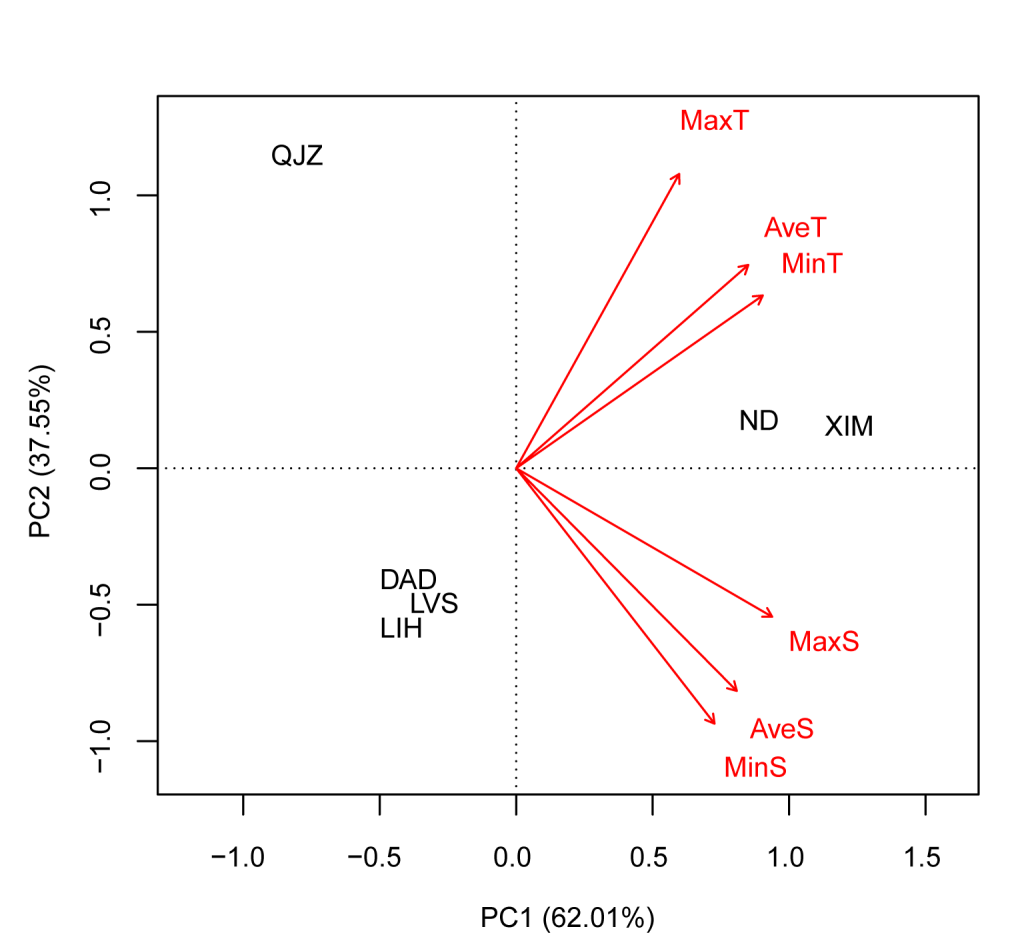

Supplement: Supplementary file 1 — Supplementary Material [file ECE3-11-4252-s001.doc]
